# Identification of key genes through the constructed CRISPR-dcas9 to facilitate the efficient production of O-acetylhomoserine in *Corynebacterium glutamicum*

**DOI:** 10.3389/fbioe.2022.978686

**Published:** 2022-09-14

**Authors:** Ning Li, Xiaoyu Shan, Jingwen Zhou, Shiqin Yu

**Affiliations:** ^1^ Science Center for Future Foods, Jiangnan University, Wuxi, China; ^2^ National Engineering Research Center of Cereal Fermentation and Food Biomanufacturing, Jiangnan University, Wuxi, China; ^3^ School of Food Science and Technology, Jiangnan University, Wuxi, China; ^4^ Engineering Research Center of Ministry of Education on Food Synthetic Biotechnology, Jiangnan University, Wuxi, China; ^5^ Jiangsu Province Engineering Research Center of Food Synthetic Biotechnology, Jiangnan University, Wuxi, China

**Keywords:** *Corynebacterium glutamicum*, l-homoserine, O-acetylhomoserine, CRISPR, *gltA*

## Abstract

O-Acetylhomoserine (OAH) is an important platform chemical for the synthesis of L-methamidophos and l-methionine. It has been produced efficiently in *Corynebacterium glutamicum*. However, a wider range of key factors had not been identified, limiting further increases in OAH production. This study successfully identified some limiting factors and regulated them to improve OAH titer. Firstly, an efficient clustered regularly interspaced short palindromic repeats/dead CRISPR associated protein 9 (CRISPR-dCas9) system was constructed and used to identify the key genes in central metabolism and branch pathways associated with OAH biosynthesis. Then, the *gltA* gene involved in TCA cycle was identified as the most critical gene. A sequential promoter P_NCgl2698_, which showed different transcriptional intensity in different strain growth periods, was used to control the expression of *gltA* gene, resulting in OAH production of 7.0 g/L at 48 h. Finally, the OAH titer of the engineered strain reached 25.9 g/L at 72 h in a 5-L bioreactor. These results show that the identification and regulation of key genes are critical for OAH biosynthesis, which would provide a better research basis for the industrial production of OAH in *C. glutamicum*.

## 1 Introduction

O-Acetylhomoserine (OAH) is an important platform compound for the synthesis of useful chemicals, such as L-methamidophos and l-methionine. The biosynthesis of OAH requires not only L-homoserine and acetyl-CoA, but also an amount of energy and reducing power. Its entire biosynthesis process involves glucose transport, glycolysis pathway, pentose phosphate pathway (PPP), tricarboxylic acid (TCA) cycle and l-aspartate metabolic pathway (Li N. et al., 2021). The TCA cycle generates NADH and ATP for OAH biosynthesis and consumes a large amount of acetyl-CoA that required for L-homoserine acetylation (Krebs, 1970). The PPP provides a large amount of NADPH for OAH biosynthesis (Wang et al., 2016). Phosphoenolpyruvate and pyruvate produced by glycolysis are precursors of l-aspartic acid biosynthesis (Piao et al., 2019) and there are many pathways to consume l-aspartic acid and acetyl-CoA (Yin et al., 2012; Milke et al., 2019; Sagong et al., 2022). Currently, regulating the expression of genes related to the L-aspartate metabolic pathway and strengthening the biosynthesis of acetyl-CoA are the main strategies to improve the production of OAH in *Corynebacterium glutamicum* and *Escherichia coli* (Wei et al., 2019; Li et al., 2022). However, these cannot further improve the titer and yield of OAH, especially in *C. glutamicum*.

Pathway design and optimization are important for the construction of high-yield strains of target products (Choi et al., 2019; Li, et al., 2020b). With the maturity of gene expression, knockout and other technologies, researchers can quickly carry out a series of modifications on chassis cells to produce new bio-based chemicals (Wei et al., 2021; Kim et al., 2022). Especially, some strategies have been applied to optimize the biosynthetic pathway composed of a large number of genes or reprogram gene expression to control complex metabolic pathways, such as the application of a multidimensional heuristic process to achieve high astaxanthin production in *E. coli* (Zhang et al., 2018), and genomic iterative substitution of large synthetic DNA fragments in *C. glutamicum* (Ye et al., 2022). As a robust industrial microorganism, the gene available regulation technology in *C. glutamicum* has been continuously created and improved, and a variety of strategies have been gradually applied (Zhang et al., 2019; Du et al., 2021). However, in addition to regulating the L-aspartate metabolic pathway, the lack of research on other pathways such as glucose transport, glycolysis and TCA cycle has hampered the strain improvement in the accumulation of OAH. Previous studies found that identifying the limiting factors of OAH biosynthesis by overexpression and knockout of a single gene in *C. glutamicum* was a very time- and labor-consuming method (Li et al., 2022), and thereby the application of a more efficient way to quickly identify the limiting step of OAH biosynthesis would be very significant.

As an efficient and accurate genome editing tool, the clustered regularly interspaced short palindromic repeats/CRISPR associated protein 9 (CRISPR-Cas9) system has been widely used in microbial metabolic engineering (Li C. et al., 2021; Qin et al., 2021; Shi et al., 2022). The functional Cas9-sgRNA complex has a high lethal rate and is unfriendly for identifying growth essential genes in prokaryotes. Therefore, two inactive mutations D10A and H840A were introduced into the RuvC1 and HNH nuclease domains of Cas9 to form dead Cas9 (dCas9), which lost its cleavage activity but still had DNA binding ability (Larson et al., 2013). This method had been widely used in many microorganisms, including *Saccharomyces cerevisiae*, *E. coli* and *C. glutamicum*. For *C. glutamicum*, previous researchers used one plasmid for dCas9 protein expression and the other plasmid for sgRNA expression to realize the application of CRISPR interference (CRISPRi) (Cleto et al., 2016; Zhang et al., 2016; Park et al., 2018). These systems consumed all two available plasmids in *C. glutamicum* (Yoon and Woo, 2018; Gauttam et al., 2019; Park et al., 2019). However, some key genes also need to be expressed by plasmids for construction of target strains. Therefore, to make CRISPRi system more widely used in *C. glutamicum*, it is necessary to construct a system using less plasmids.

In this study, to systematically identify the key genes for efficient biosynthesis of OAH in *C. glutamicum*, two sites of *cas9* gene in the genome were mutated to form dCas9 protein, based on the CRISPR-Cas9 system constructed in our previous study (Li N. et al., 2021). Taking glucose transport, glycolysis pathway, PPP and TCA cycle as the targets, the CRISPR-dCas9 system was used to inhibit the expression of related genes. It was found that the genes related to glucose transport, glycolysis and TCA cycle had a significant impact on the OAH accumulation. Finally, the sequential promoter was used to regulate *gltA* gene, which improved the OAH titer and provided some useful information for the efficient biosynthesis of OAH in *C. glutamicum*.

## 2 Materials and methods

### 2.1 Strains and plasmids

The *C. glutamicum* ATCC 13032 variants were used for the biosynthesis of target products. *E. coli* JM109 was used for plasmid construction. The plasmid pEC-XK99E and pXMJ19 were used for gene expression, pXMJ19ts was used for sgRNA expression, pK18mobsacB was used for genome editing. Details are listed in Tables 1, 2.

**TABLE 1:** Strains used in this study.

Strain	Description	Source
*E. coli* JM109	Plasmid amplification	Invitrogen
*C. glutamicum* ATCC 13032	Wild-type	ATCC
Cg17	13,032 derivative, ∆*NCgl1021::P*_*tuf*_ *-cas9::P*_*prp*_ *-recET*, *∆mcbR*, *∆metD*, *∆thrB*, *∆NCgl2360::P*_*sod*_ *-thrA* ^*S345F*^, *∆NCgl2688::P*_*NCgl1676*_ *-metX* ^*r*^ *_Lm*, *∆metY*, *∆pck::P*_*sod*_ *-aspC*, *P*_*sod*_ *-pyc* ^*P458S*^, *P*_*sod*_ *-lysC* ^*T311I*^, *P*_*sod*_ *-asd*, *P*_*sod*_ *-hom* ^*V59A*^, *P*_*sod*_ *-brnFE*, *icd* ^*M1V*^, *dapA* ^*M1V*^	Li et al. (2022)
Cg17-3	Cg-17 harboring pEC-*thrA* ^*S345F*^-P_NCgl1676_-*metX* ^*r*^	Li et al. (2022)
Cg18	D10A and H840A of Cas9 in Cg17	This study
Cg18-(T)	Cg18 harboring pXM-sgRNAi-*mCherry*(T) and pNCgl1676-*mCherry*	This study
Cg18-null(T)	Cg18 harboring pXM-sgRNAi-Null and pNCgl1676-*mCherry*	This study
Cg18-1	D10A and H840A of Cas9 in Cg17-3	This study
Cg18-1 (null)	Cg18-1 harboring pXM-sgRNAi-Null	This study
Cg18-1(X)	Cg18-1 harboring pXM-sgRNAi-X	This study
Cg19-∆*gapA*	∆*gapA* in the strain Cg17	This study
Cg19-∆*gltA*	∆*gltA* in the strain Cg17	This study
Cg20	∆*aspA::P*_*tuf*_ *-aspB_Pa* in the strain Cg17	This study
Cg20-1	Cg-20 harboring pEC-*thrA* ^*S345F*^-P_NCgl1676_-*metX* ^*r*^	This study
Cg20-2	∆*aspA::P*_*tuf*_ *-aspB_Pa* in the strain Cg17-3	This study
Cg21	∆*aspA::P*_*tuf*_ *-aspB_Pa*, ∆*gltA* in the strain Cg17	This study
Cg21-1	Cg21 harboring pXMJ19 and pEC-XK99E	This study
Cg21-2	Cg21 harboring pXMJ19 and pEC-*thrA* ^*S345F*^-P_NCgl1676_-*metX* ^*r*^	This study
Cg21-3	Cg21 harboring pXM-*aspB*_Pa and pEC-*thrA* ^*S345F*^-P_NCgl1676_-*metX* ^*r*^	This study
Cg22	∆*aspA::P*_*tuf*_ *-aspB_Pa*, ∆*gltA::P*_*NCgl2698*_-*gltA* in the strain Cg17	This study
Cg22-1	Cg22 harboring pEC-*thrA* ^*S345F*^-P_NCgl1676_-*metX* ^*r*^	This study
Cg23-1	∆*Cas9*, ∆*recET* in the strain Cg22-1	This study

**TABLE 2:** Plasmids used in this study.

Plasmid	Description	Source
pEC-XK99E	IPTG-inducible P_trc_ promoter, Km^r^	Kirchner and Tauch, (2003)
pXMJ19	IPTG-inducible P_tac_ promoter, Cm^r^	Jakoby et al. (1999)
pK18mobsacB	Suicide vector, Km^r^	Schafer et al. (1994)
pEC-*thrA* ^*S345F*^-P_NCgl1676_-*metX* ^*r*^	pEC-XK99E carrying *thrA* ^*S345F*^ and *metX* ^*r*^, *metX* ^*r*^ under the control of P_NCgl1676_ promoter	Li et al. (2022)
pK18-dCas9-D10A	Mutate the 10th amino acid site (aspartate to alanine) of Cas9	This study
pK18-dCas9-H840A	Mutate the 840th amino acid site (histidine to alanine) of Cas9	This study
pP_trc_sgRNA-dCas9	Mutate the 10th amino acid site (aspartate to alanine) of Cas9	This study
pP_H36_sgRNA-dCas9	Mutate the 840th amino acid site (histidine to alanine) of Cas9	This study
pP_trc_P_H36_sgRNA-dCas9	Mutate the 10th (aspartate to alanine) and 840th amino acid site (histidine to alanine) of Cas9	This study
pNCgl1676-*mCherry*	The plasmid carrying the *mCherry* gene under the control of the promoter P_NCgl1676_	Li, et al. (2020a)
pXM-sgRNAi-*mCherry*(T)	Expression of sgRNA localized to the template chain of *mCherry* gene	This study
pXM-sgRNAi-Null	Expression of sgRNA without localization site	This study
pXM-sgRNAi-X	Expression of sgRNA localized to X gene	This study
pK18-P_tuf_-aspB_Pa	Integrated expression of *aspB* gene from *Pseudomonas aeruginosa*	This study
pK18-gltA-QC	Deletion of *gltA* gene	This study
pK18-P_NCgl2698_-gltA	Replace native promoter with promoter P_NCgl2698_ of *gltA* gene	This study
pP_trc_P_H36_sgRNA-recET-cas9	Inactivation of *recET* and *cas9* genes	This study

### 2.2 Strains culture

The strains culture conditions were described as our previous study (Li et al., 2022). For the shake flask culture, the engineered *C. glutamicum* strains were precultured in seed medium, cultured in fermentation medium with 4% inoculum. The fermentation medium was supplemented with 5 g/L acetate at 24 and 36 h, respectively. All strains were grown at 30°C and 220 rpm in shaking incubators. For a 5-L bioreactor culture, the strain was precultured in the seed medium, cultured in the fermentation medium with 10% inoculum. The pH was adjusted to 6.0 with 50% ammonia. Through ventilation and stirring speed, the dissolved oxygen (DO) was maintained at about 25%. For *E. coli*, the working concentration of kanamycin was 50 mg/L and that of chloramphenicol was 25 mg/L. For *C. glutamicum*, the working concentration of kanamycin was 15 mg/L and that of chloramphenicol was 7.5 mg/L.

### 2.3 Construction of Cas9 inactivated variant and genome editing plasmids

The plasmids pK18-dCas9-D10A and pK18-dCas9-H840A were used to mutate the 10th amino acid site from aspartate to alanine and 840th amino acid site from histidine to alanine of Cas9 through the sucrose lethal method (Tan et al., 2012). The plasmids pP_trc_sgRNA-dCas9 and pP_H36_sgRNA-dCas9 were also used to mutate the two sites through the CRISPR-Cas9 system. The plasmid pP_trc_P_H36_sgRNA-dCas9 was used to mutate the two sites simultaneously through the CRISPR-Cas9 system. The primers are listed in Supplementary Table S1.

### 2.4 Construction of sgRNA expression plasmids

To inhibit the expression of candidate genes using the CRISPR-dCas9 system, a series of sgRNA expression plasmids were constructed. The primers are listed in Supplementary Table S2. The DNA fragment P_glyA_2 was amplified by polymerase chain reaction (PCR) with *C. glutamicum* ATCC 13032 genome as template and P_glyA_2-F/P_glyA_2-R as primers. The DNA fragments X-sgRNA-BIRI were amplified with plasmid pHAsgRNA as template and X-sgRNA-F/BIRI2-R as primers, respectively (Li N. et al., 2021). The plasmid pXMJ19 was digested with DNA restriction enzyme *Apa* I and *BamH* I to generate DNA fragment pXMJ19-AB. The DNA fragments, including pXMJ19-AB, P_glyA_2 and X-sgRNA-BIRI, were then assembled by Gibson assembly to generate the plasmids pXM-sgRNAi-X corresponding to each candidate gene X.

### 2.5 Real-time quantitative PCR

Like the fermentation culture, the strains were cultured in the fermentation medium. After 24 h, the strains were collected and washed briefly by diethylpyrocarbonate-treated water (without RNase), then quickly cooled with liquid nitrogen. The RNA extraction kit, cDNA transcription kit and SYBR Premix Ex Taq II were purchased from TaKaRa (Dalian, China). The qPCR was performed on a LightCycler 480 II Real-time PCR instrument (Roche Applied Science, Mannheim, Germany). The 16s rDNA was the served as the house-keeping gene. The 2^−ΔΔCt^ method was used to determine the relative expression levels of genes (Chen et al., 2022). The primers are listed in Supplementary Table S3.

### 2.6 Delete the *cas9* and *recET* genes in the genome

To delete the *cas9* and *recET* genes, the donor DNA in the plasmid pP_trc_P_H36_sgRNA-dCas9 was replaced by DNA fragment cas9-recET to generate plasmid pP_trc_P_H36_sgRNA-recET-cas9. For the donor DNA, the upstream DNA fragment of plasmid pP_trc_P_H36_sgRNA-recET-cas9 was the same as that of plasmid pP_trc_P_H36_sgRNA-dCas9. The downstream DNA fragment was amplified with recEC-cas9-DOWN-F/recE-Ccas9-DOWN-R as primers and the genome of strain Cg17 as the template. The plasmid pP_trc_P_H36_sgRNA-dCas9 was linearized through PCR with XXH-F/XXH-R as the primers. The primers are listed in Table S1.

### 2.7 The transformation methods for *C. glutamicum*

About 1 μg of plasmid was transformed into the competent *C. glutamicum* by electric shock with 1 mm shock cup and 1800 V voltage. The strains transferred to the LBHIS medium and incubated at 46°C for 6 min, then incubated at 30°C for 1–3 h. Next, the strains were cultured on the LBHIS plate containing the corresponding antibiotics at 30°C for 3 days. The positive transformants were verified by colony PCR with the corresponding primers. For the sucrose lethal method, the positive transformants with appropriate dilution were cultured on the LBHIS plate containing 100 g/L of sucrose at 30°C for 3 days. Finally, the following positive transformants were verified by colony PCR with the corresponding primers.

### 2.8 Analytical methods

#### 2.8.1 Detection of fluorescence intensity and cell concentration

The optical density (OD_600_) and intensity of fluorescent strains were measured with a Synergy H1 Hybrid Multi-Mode Reader (BioTek Instruments, Winooski, VT). For red fluorescence determination, the emission wavelength was 587 nm and the excitation wavelength was 610 nm.

#### 2.8.2 Determination of biomass

The fermentation broth was diluted appropriately with 0.1 M HCl to dissolve the remaining CaCO_3_ and the OD_600_ was determined by a Biophotometer D30 (Eppendorf AG, Hamburg).

#### 2.8.3 Determination of glucose and acetic acid concentrations

The concentration of glucose and acetic acid in fermentation broth were determined by high performance liquid chromatography (HPLC) with a refractive-index detector and Aminex HPX-87H column (Bio-Rad, Richmond, CA).

#### 2.8.4 Determination of amino acid concentration

After centrifugation, the supernatant of the fermentation broth was diluted with 0.4 M trichloroacetic acid, then saved at 4°C for more than 4 h. The pretreatment samples were centrifuged at 4°C for 10 min to take the supernatant, then diluted with 0.1 M acetic acid and filtered with a 0.22 µm membrane filter. The concentration of amino acids was determined after precolumn derivatization (Piao et al., 2019).

## 3 Results and discussions

### 3.1 Construction of an O-acetylhomoserine-producing strain

**FIGURE 1:**
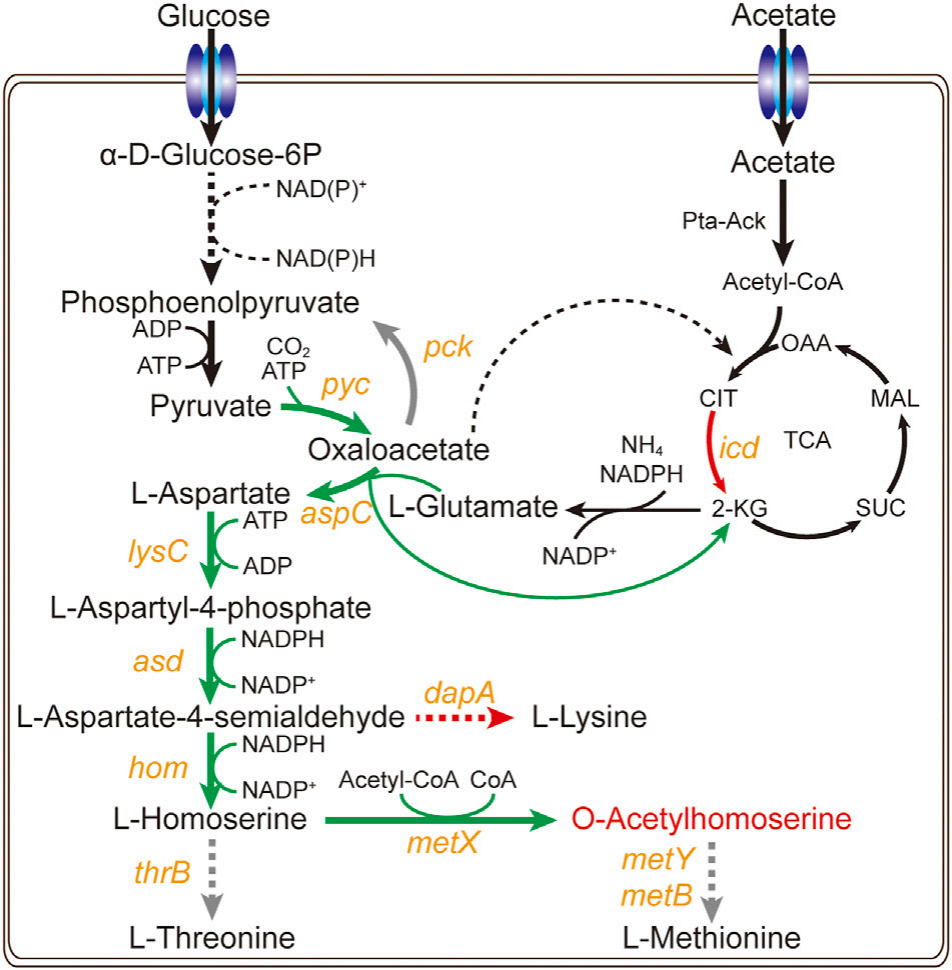
Metabolic engineering strategy for construction of the OAH-producing strains. Yellow italics represent genes. The black arrows represent the unmodified pathways, the grey arrows represent the knockout pathways, the green arrows represent the up-regulated pathways, the red arrows represent the down-regulated pathways.

The bacterium *C. glutamicum* ATCC 13032 cannot naturally accumulate L-homoserine and O-acetylhomoserine. As in our previous studies (Li et al., 2020c; Li N. et al., 2021), the strong promoter P_sod_ was used to enhance the expression of key genes in L-homoserine biosynthesis pathway, including *pyc*, *lysC*, *asd*, hom, and *brnFE* (a nonspecific transporter component). The *aspC* gene and *thrA* ^*S345F*^ gene from *E. coli* was then introduced into the genome, and the expression of *icd* and *dapA* genes was downregulated through replacing the start codon ATG with GTG. The key genes of competition and degradation pathways including *mcbR*, *thrB*, *metB*, and *metY* were knocked out to obtain a L-homoserine-producing strain. The exogenous l-homoserine acetyltransferase variant MetX^r^ from *Leptospira meyeri* was introduced to obtain an engineered strain Cg17 (Figure 1), which accumulated 0.98 g/L of OAH after 48 h. To drain pyruvate flow to the L-aspartate biosynthesis pathway, reduce the use of pyruvate for the acetyl-CoA biosynthesis and promote the OAH accumulation, the expression of bifunctional L-aspartokinase and L-homoserine dehydrogenase (ThrA^S345F^) from *E. coli* and MetX^r^ were enhanced through the plasmid pEC-XK99E to generate strain Cg17-3 (Li et al., 2022). With the addition of 5 g/L acetate at 24 and 36 h, the resulting strain Cg17-3 produced 5.2 g/L of OAH at 48 h. Although the *C. glutamicum* achieved efficient OAH biosynthesis, few of the limiting factors of OAH biosynthesis were identified. Therefore, to further improve the OAH accumulation, expanding the identification range of key genes was a very worthwhile work.

### 3.2 Construction and evaluation of CRISPR-dCas9 in *C. glutamicum*

**FIGURE 2:**
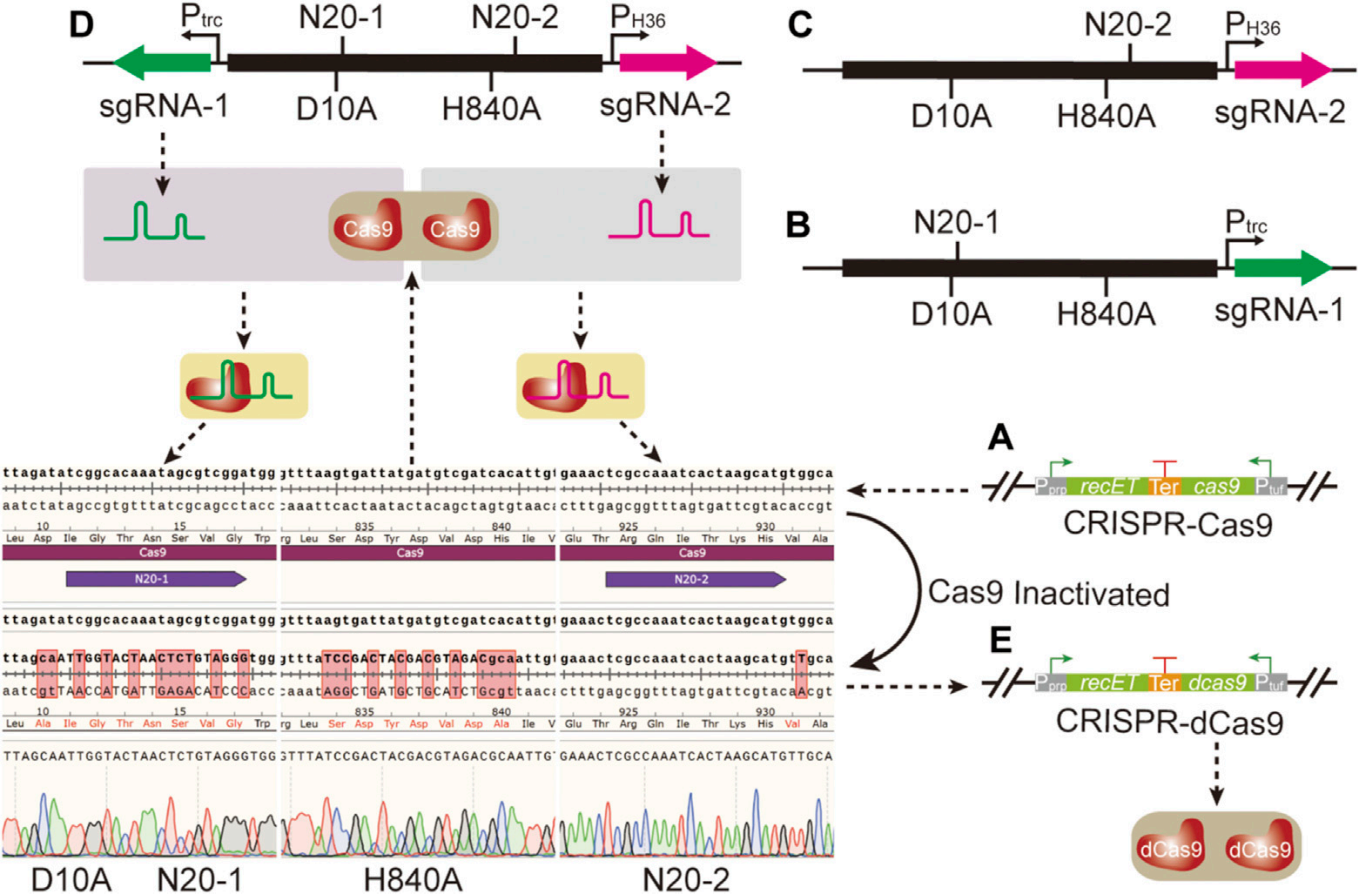
Site directed mutation of *cas9* gene through CRISPR-Cas9 system. **(A)** The CRISPR-Cas9 system. **(B)** The plasmid pP_trc_sgRNA-dCas9; this plasmid was used to mutate the 10th amino acid site from aspartate to alanine of Cas9. **(C)** The plasmid pP_H36_sgRNA-dCas9; this plasmid was used to mutate the 840th amino acid site from histidine to alanine of Cas9. **(D)** The plasmid pP_trc_P_H36_sgRNA-dCas9; this plasmid was used to mutate the 10th amino acid site from aspartate to alanine and 840th amino acid site from histidine to alanine of Cas9 simultaneously. **(E)** The CRISPR-dCas9 system.

To construct a CRISPR-dCas9 system, two mutation sites D10A and H840A were introduced into the *cas9* gene that had been integrated into the genome in our previous studies (Figure 2A) (Li N. et al., 2021). The conventional genome editing method based on SacB was selected to mutate the two sites of Cas9 through two iterative editing. However, the whole process was time- and labor-consuming due to the required two edits, which made the rapid switching from CRISPR-Cas9 system to CRISPR-dCas9 system impossible. To facilitate the application of CRISPR-dCas9 in subsequent studies, CRISPR-Cas9 system was used to mutate Cas9. Two N20 (N20-1 and N20-2) located in distinct positions on *cas9* gene were designed, generating sgRNA-1 and sgRNA-2. Two sgRNA coding sequences were inserted into pXMJ19ts respectively, together with donor DNA containing two mutation sites to obtain pP_trc_sgRNA-dCas9 and pP_H36_sgRNA-dCas9 (Figures 2B,C). When the two plasmids were transformed into strain Cg17 respectively, only one site mutation could be achieved. Therefore, two sgRNA expression frames were inserted into pXMJ19ts simultaneously, together with donor DNA containing two mutation sites to obtain plasmid pP_trc_P_H36_sgRNA-dCas9 (Figure 2D). After the plasmid was transformed into strain Cg17, the strain Cg18 with two mutation sites was obtained (Figure 2E), which achieved the rapid switching from CRISPR-Cas9 to CRISPR-dCas9 as shown in Figure 2.

**FIGURE 3:**
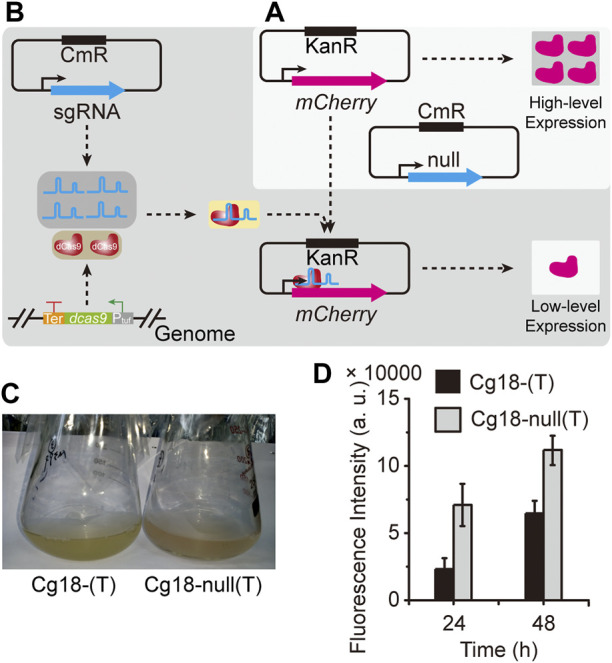
Evaluation of the CRISPR-dCas9 system. The gene inhibition ability of CRISPR-dCas9 system was evaluated through the expression intensity of mCherry protein. **(A)** The high-level expression of *mCherry* gene. **(B)** The CRISPR-dCas9 system was used to inhibit *mCherry* gene expression. **(C)** The strains with *mCherry* gene expression plasmids; the strain Cg18-(T) harbored pXM-sgRNAi-*mCherry*(T) and pNCgl1676-*mCherry*; the strain Cg18-null(T) harbored pXM-sgRNAi-Null and pNCgl1676-*mCherry*. **(D)** The Fluorescence intensity of the strains harboring *mCherry* gene expression plasmids. Error bars are standard deviation of triplicate experiments.

To evaluate the CRISPR-dCas9 system constructed above, the expression plasmid pNCgl1676-*mCherry* containing *mCherry* gene and pXM-sgRNAi-*mCherry*(T) containing sgRNA targeting the template chain of *mCherry* gene were transformed into Cg18, generating the strain Cg18-(T). The strain Cg18-null(T) containing pNCgl1676-*mCherry* and pXM-sgRNAi-Null without localization site was used as the control. As shown in Figure 3, CRISPR-dCas9 system had a strong inhibitory effect on *mCherry* gene expression at 24 and 48 h. The fluorescence intensity of strain Cg18-(T) was 67.6 and 36.6% of Cg18-null(T), respectively.

For *C. glutamicum*, it is very difficult and time-consuming to identify the key genes by classical gene knockout or overexpression (Yang et al., 2016). The CRISPRi can inhibit gene expression without destroying the genome. In previous studies, the CRISPR-dCas9 system had been constructed and applied in *C. glutamicum*. However, dead *cas9* (*dcas9*) gene and sgRNA consumed two antibiotic resistant plasmids pEC-XK99E and pXMJ19, resulting in no plasmid for the overexpression of other genes (Cleto et al., 2016). Therefore, Zhang et al. introduced *dcas9* gene and sgRNA expression frames into one plasmid and another plasmid can be used to express other target genes (Zhang et al., 2016). However, like Cas9, high level expression of dCas9 would also have an adverse effect on the growth of *C. glutamicum*. Some studies found that dCas9-sgRNA targeting the template chain had no obvious inhibitory effect on the gene expression (Larson et al., 2013), while some studies showed a strong inhibitory effect (Bikard et al., 2013). For *C. glutamicum*, dCas9-sgRNA had a strong inhibitory effect on gene expression whether targeted the template or non-template chains (Cleto et al., 2016; Zhang et al., 2016). In this study, the dCas9-sgRNA targeted the template chain of the *mCherry* gene, resulting in the down-regulation of *mCherry* expression. These indicated that this CRISPR-dCas9 system using a single plasmid had a strong ability to inhibit gene expression.

### 3.3 Application of CRISPR-dCas9 for identification of the key genes

**FIGURE 4:**
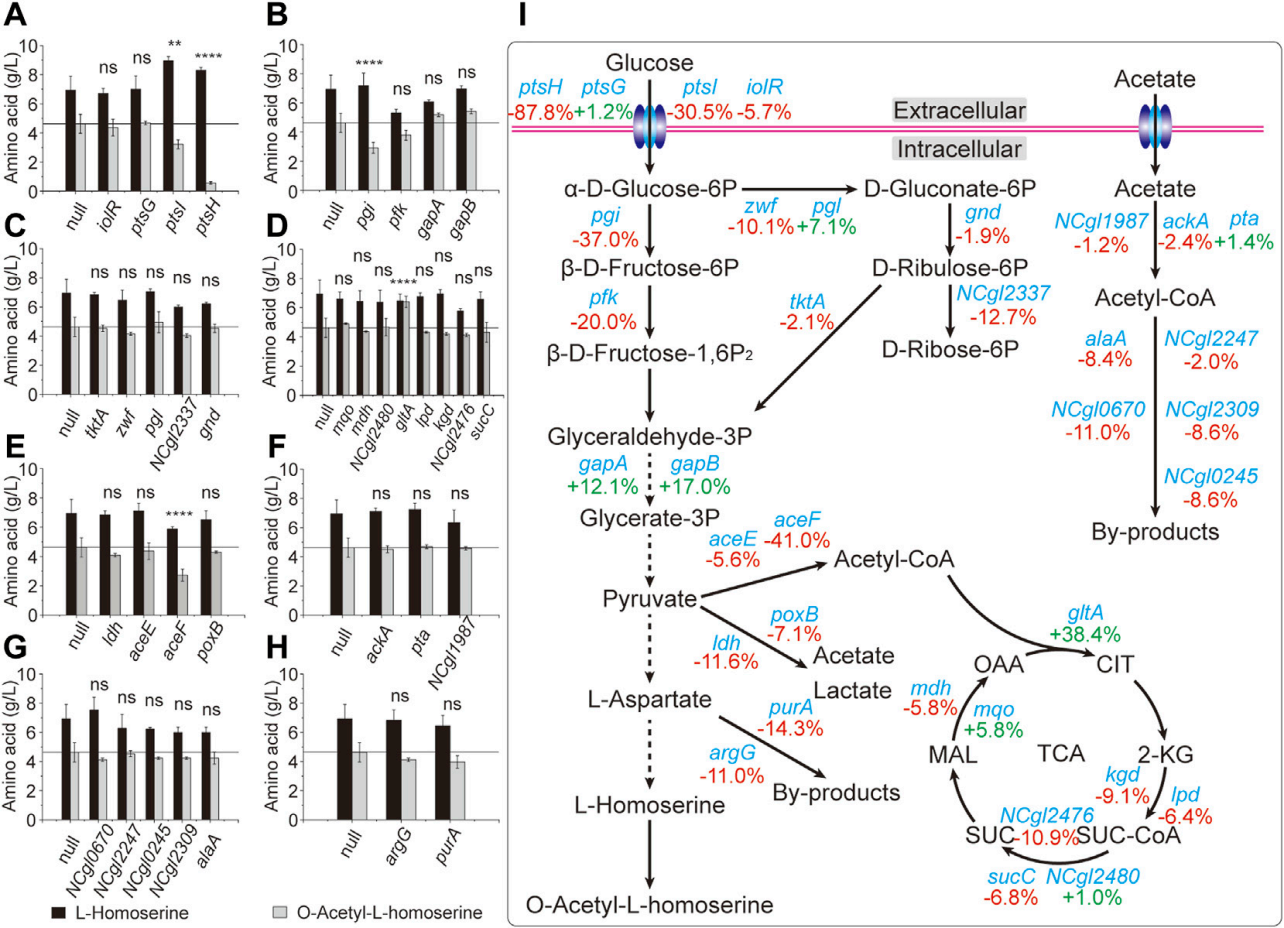
Identification of the key genes through the CRISPR-dCas9 system. The results of inhibition of different genes. **(A)** The genes of glucose transport. **(B)** The genes of glycolytic pathway. **(C)** The genes of pentose phosphate pathway. **(D)** The genes of TCA cycle. **(E)** The genes of pyruvate catabolism. **(F)** The genes of acetate acylation. **(G)** The genes of acetyl-CoA utilization. **(H)** The genes of l-aspartic acid degradation and metabolism. **(I)** The overall schematic diagram. Error bars are standard deviation of triplicate experiments. Statistical significance was determined with 95% confidence (ns > 0.05, *p < 0.05, **p < 0.005, ***p < 0.0005, ****p < 0.0001).

**FIGURE 5:**
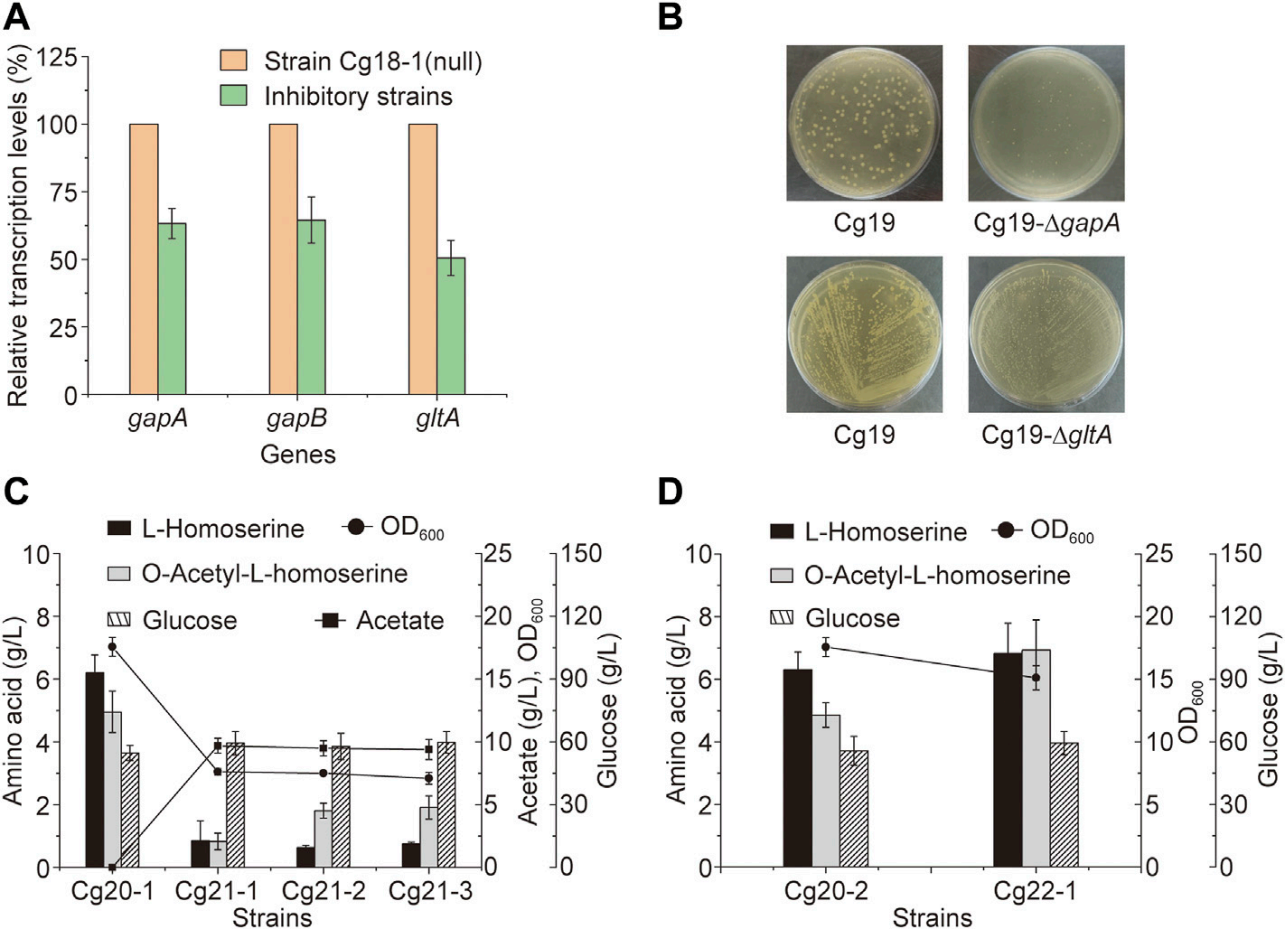
Metabolic engineering of the key genes. **(A)** The transcriptional levels of *gapA*, *gapB* and *gltA* genes under the inhibition of the CRISPR-dCas9 system, the control strain was strain Cg18-1 (null). **(B)** The growth state of the strains on the plates after knockout of *gapA* or *gltA* genes at 72 h. **(C)** The OAH titer after the knockout of *gltA* gene and the heterologous expression of a NADH dependent aspartate aminotransferase. **(D)** The OAH titer after the expression of *gltA* gene under the control of P_NCgl2698_ promoter. Error bars are standard deviation of triplicate experiments.

The main pathways involved in the OAH biosynthesis include glucose transport, glycolysis, PPP, TCA cycle and l-aspartate metabolic pathway (Li et al., 2017). However, many related studies have only focused on the l-aspartate metabolic pathway, which made the metabolic engineering to construct a more efficient OAH-producing strain less targeted (Jin and Bao, 2021). Therefore, the rapid identification of the limiting steps in the OAH biosynthesis was of great significance for subsequent metabolic engineering. Some important candidate genes from these related pathways were selected (Goettl et al., 2021; Krahn et al., 2021), including *ptsG*, *ptsH*, *ptsI*, *iolR* of glucose transport; *pgi*, *pfk*, *gapA*, *gapB* of glycolytic pathway; *zwf*, *tktA*, *pgl*, *gnd*, *NCgl2337* of the PPP; *gltA*, *mdh*, *mqo*, *kgd*, *lpd*, *sucC*, *NCgl2476*, *NCgl2480* of the TCA cycle; *aceE*, *aceF*, *ldh*, *poxB* of pyruvate catabolism; *ackA*, *pta*, *NCgl1987* of acetate acylation; *alaA*, *NCgl2247*, *NCgl0670*, *NCgl2309*, *NCgl0245* of acetyl-CoA utilization and *purA*, *argG* of l-aspartic acid degradation and metabolism. To identify the relationship between these candidate genes and OAH biosynthesis, N20 sequences located in these candidate genes were designed to generate respective sgRNA expression plasmids. Like the strain Cg17, the *cas9* gene of the OAH-producing strain Cg17-3 was mutated to generate strain Cg18-1. The sgRNA expression plasmids were transformed into strain Cg18-1 to obtain strains Cg18-1(X), respectively. The strain Cg18-1 (null) containing pXM-sgRNAi-Null without localization site was used as the control strain. As shown in Figure 4, the OAH titer decreased after the expression of many genes was inhibited. Some of them increased the OAH titer, including *gltA* (38.4%), *gapA* (12.1%) and *gapB* (17.0%) (Figures 4B,D). However, only the OAH titer after *gltA* gene inhibition was significant. The results showed that the transcription levels of *gapA*, *gapB* and *gltA* genes of the inhibitory strains at 24 h were 63.2, 64.5, and 50.5% of those non-inhibitory strains, respectively (Figure 5A).

*C. glutamicum* contains two glyceraldehyde-3-phosphate dehydrogenases, including GapA and GapB. GapA is dependent on NADH, and GapB is dependent on NADPH (Takeno et al., 2016). For the OAH biosynthesis, excessive NADH would be generated and a large amount of NADPH would be consumed. We speculated that the inhibition of *gapA* and *gapB* genes by CRISPR-dCas9 may interfere the biosynthesis of NADH and NADPH. However, when the *gapA* gene of strain Cg17 was knocked out to generate strain Cg19-∆*gapA*, which grew very slowly on the screening medium (Figure 5B), and cannot be used for subsequent metabolic engineering (Takeno et al., 2010). After the *gapB* gene was knocked out, the growth of engineered strain was not affected, but the OAH production did not change. More detailed reasons need to be further explored. Unlike the *gapA* and *gapB* genes, the expression level of *gltA* gene determines the TCA cycle rate and l-homoserine acetylation rate, the flow of acetyl-CoA into the TCA cycle is a key node of OAH accumulation. After the *gltA* gene, which encodes citrate synthase, was inhibited, the OAH titer increased to 6.4 g/L, which was the best among all the gene inhibitions, and the *gltA* gene should be a key candidate gene for the further regulation.

### 3.4 The expression of *gltA* gene under the control of conditional promoter

Down-regulation of *gltA* gene expression through the CRISPR-dCas9 system can enhance the OAH accumulation. Therefore, the *gltA* gene of strain Cg17 was knocked out, and the mutant strain Cg19-*∆gltA* was generated. However, the Cg19-∆*gltA* showed poor growth, which made it impossible to conduct subsequent metabolic engineering (Figure 5B). It was speculated that the knockout of *gltA* gene cannot provide adequate α-ketoglutarate used for l-glutamate biosynthesis (Li X. et al., 2021), resulting in the inefficient conversion of oxaloacetate to L-aspartate using glutamate as an ammonia donor and the slow metabolic rate of the L-lysine biosynthesis pathway (Jeong et al., 2019). Meso-2,6-diaminoheptanedioate, one intermediate metabolite of L-lysine biosynthesis, was the precursor of peptidoglycan biosynthesis and the insufficient supply of peptidoglycan would lead to slow strain growth (Sgobba et al., 2018).

To enhance the cell growth, a NADH dependent aspartate aminotransferase, encoded by *aspB* gene from *Pseudomonas aeruginosa* (Wu et al., 2018), was expressed in the genome of strain Cg17 to generate Cg20. The strain Cg20 relieved the over-dependence of transamination on l-glutamate. Indeed, the growth of strain Cg21 (the strain Cg20 with *gltA* gene inactivation) was greatly restored. Then, three plasmid combinations including pXMJ19 and pEC-XK99E, pXMJ19 and pEC-*thrA* ^*S345F*^-P_NCgl1676_-*metX* ^*r*^, pXM-*aspB*_Pa and pEC-*thrA* ^*S345F*^-P_NCgl1676_-*metX* ^*r*^ were transformed into strain Cg21, resulting in strains Cg21-1, Cg21-2 and Cg21-3, respectively. As shown in Figure 5C, when the *gltA* gene was knocked out, all the biomass, l-homoserine titers (0.86 g/L, 0.63 g/L, and 0.75 g/L) and OAH titers (0.83 g/L, 1.81 g/L, and 1.91 g/L) decreased sharply, and acetic acid was barely consumed.

The strain needs a large amount of NADH, ATP and some important metabolites through TCA cycle for the biosynthesis of primary metabolism to meet the strain growth, the *gltA* gene inactivation has a very adverse impact on the strain (Xu et al., 2018). Therefore, a strong *gltA* gene expression level is required in the early stage of cell growth. The previous study showed that the transcriptional intensity of promoter P_NCgl2698_ reduced from 75 to 8% of the constitutive strong promoter P_tuf_ after entering the stable growth period (Ma et al., 2018). It was used to replace the native promoter to regulate the expression of *gltA* gene in Cg20, generating strain Cg22. Then, the plasmid pEC-*thrA* ^*S345F*^-P_NCgl1676_-*metX* ^*r*^ was introduced into Cg22 to generate strain Cg22-1. Taking strain Cg20-2 as the control, which was obtained from Cg17-3 with the integrated expression of the *aspB*_*Pa* gene, the OAH titer increased by 45.8% to 7.0 g/L (Figure 5D). Besides, the growth of strain Cg22-1 was restored compared to strain Cg21-3. Therefore, using P_NCgl2698_ to control the expression of *gltA* gene could enhance the OAH accumulation, but did not have a significant impact on the growth of bacteria. The results showed that further optimization of *gltA* gene expression could enhance OAH biosynthesis.

### 3.5 The production of OAH in a 5-L bioreactor

**FIGURE 6:**
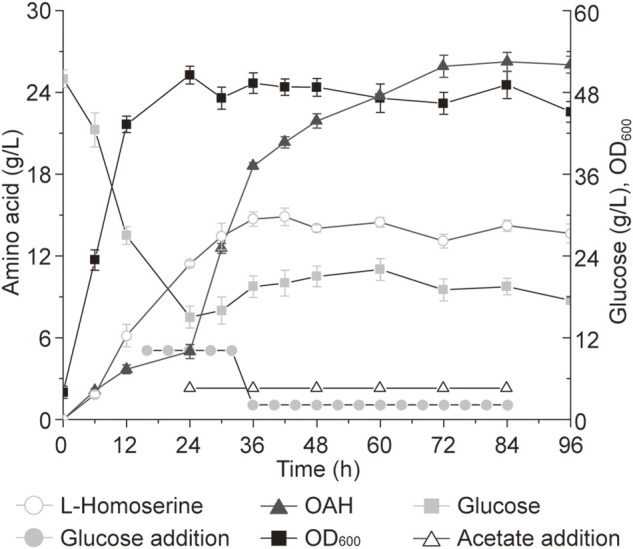
The titer of OAH in 5-L bioreactor. In a dissolved oxygen (DO) at 25% and pH 6.0, OAH can be biosynthesized quickly with glucose addition in a 5-L bioreactor. Error bars are standard deviation of triplicate experiments.

The continuous and stable supply of substrate and pH control has a great impact on the production capacity, but these are difficult to achieve in a shake flask (Tan et al., 2019). Therefore, a 5-L bioreactor was used to further explore the production potential of high-performance strains. Before that, the *cas9* and *recET* genes in the genome of strain Cg22-1 were inactivated using the plasmid pP_trc_P_H36_sgRNA-recET-cas9 through the CRISPR-Cas9 system to obtain strain Cg23-1. According to previous studies, the addition of acetate was key for the efficient biosynthesis of OAH in *C. glutamicum* (Li et al., 2022). As shown in Figure 6, to make more acetyl-CoA produced from acetic acid for OAH biosynthesis, 5 g/L of acetic acid was added every 12 h starting from 24 h. At the same time, when the total consumption concentration of glucose reached 100 g/L, 500 g/L glucose was used to maintain the glucose concentration at 10–20 g/L. After the total glucose consumption exceeded 100 g/L, the mixture of 500 g/L glucose and 120 g/L ammonium sulfate was used to maintain the glucose concentration at 10–20 g/L. Finally, the OAH titer of strain Cg23-1 reached 25.9 g/L at 72 h, which was about 48.9% higher than the previous study (Li et al., 2022). This is the highest OAH titer in *C. glutamicum*, which proves that it has great potential.

## 4 Conclusions

Based on previous work, the OAH concentration increased to 5.2 g/L through the enhancement of the OAH biosynthesis pathway genes and acetate supplementation in *C. glutamicum*. Then, the rapid switching from CRISPR-Cas9 to CRISPR-dCas9 was achieved through the previously constructed CRISPR-Cas9 system. Using the CRISPR-dCas9 system, the *gltA* gene was identified as the key factor affecting the OAH accumulation. The *gltA* gene expression was controlled by the sequential promoter P_NCgl2698_, resulting in the increase of OAH accumulation. Finally, the OAH titer of the engineered strain reached 25.9 g/L at 72 h in a 5-L bioreactor. This was the highest OAH titer in *C. glutamicum* and would provide a good reference for the industrial production of OAH.

## Data Availability

The original contributions presented in the study are included in the article/Supplementary Material, further inquiries can be directed to the corresponding author.
