# Supplementary material for: Identification of key genes through the constructed CRISPR-dcas9 to facilitate the efficient production of O-acetylhomoserine in Corynebacterium glutamicum
Source: Front Bioeng Biotechnol. 2022 Sep 14;10:978686. doi: 10.3389/fbioe.2022.978686 (PMC9515461; doi:10.3389/fbioe.2022.978686)
Supplement: Supplementary file 1 [file DataSheet1.pdf]

Supporting Information

Identification of key genes through the constructed CRISPR-dcas9 to facilitate the efficient production of O-acetylhomoserine in *Corynebacterium glutamicum*

Ning Li[1,2,3], Xiaoyu Shan[1,2], Jingwen Zhou[1,2,4,5], Shiqin Yu[1,2*]

[1] Science Center for Future Foods, Jiangnan University, 1800 Lihu Road, Wuxi, Jiangsu 214122, China;

[2] National Engineering Research Center of Cereal Fermentation and Food Biomanufacturing, Jiangnan University, 1800 Lihu Road, Wuxi, Jiangsu 214122, China;

[3] School of Food Science and Technology, Jiangnan University, 1800 Lihu Road, Wuxi, Jiangsu 214122, China;

[4] Engineering Research Center of Ministry of Education on Food Synthetic Biotechnology, Jiangnan University, 1800 Lihu Road, Wuxi, Jiangsu 214122, China;

[5] Jiangsu Province Engineering Research Center of Food Synthetic Biotechnology, Jiangnan University, 1800 Lihu Road, Wuxi, Jiangsu 214122, China.

[*] Corresponding author

Shiqin Yu

Mailing address: Science Center for Future Foods, Jiangnan University, 1800 Lihu Road, Wuxi, Jiangsu 214122, China.

Phone: +86-510-85914371, Fax: +86-510-85914371

E-mail: shiqin.yu@jiangnan.edu.cn

Supporting tables

Table S1. The primers used for construction of the genome editing plasmids.

Primer	Primer sequence (5'-3')	Function
dcas9-UP1-F	cgacggccagtgccaagcttaacacccagagcctgtcgc	pK18-dCas9-D10A
dcas9-UP1-R	CCCTACAGAGTTAGTACCAATtgctaagcctattgagtatttcttatccataatc	
dcas9-DOWN1-F	gcaATTGGTACTAACTCTGTAGGGtgggcggtgatcactgatga	
dcas9-DOWN1-R	agctcggtacccggggatccccacttgcgttaatagggttttct	
D10A-JL-R	CCCTACAGAGTTAGTACCAATtg	
dcas9-UP2-F	cgacggccagtgccaagcttgtcgccgttatactggttggg	pK18-dCas9-H840A
dcas9-UP2-R	tgcGTCTACGTCGTAGTCGGAtaaacgattaatatctaattcttggtcc	
dcas9-DOWN2-F	TCCGACTACGACGTAGACgcaattgttccacaaagtttccttaaag	
dcas9-DOWN2-R	agctcggtacccggggatcctcaccatagacaaactccgattca	
H840A-JL-F	CGACTACGACGTAGACgca	
19ts-XXH-F	GATCCCCGGGTACCGAGCT	pP_{trc}sgRNA-dCas9
19ts-XXH-R	CTGTTGCCCGTCTCACTGGTG	

dCas9-UP3-F	caccagtgagacgggcaacagtaaacacccagagcctgtcgc	
dcas9-UP1-R	CCCTACAGAGTTAGTACCAATtgctaagcctattgagtatttcttatccataatc	
dCas9-Middle-F	gcaATTGGTACTAACTCTGTAGGGtg	
dcas9-UP2-R	tgcGTCTACGTCGTAGTCGGAtaaacgattaatatctaattcttggtcc	
dcas9-DOWN2-F	TCCGACTACGACGTAGACgcaattgttccacaaagtttccttaaag	
dCas9-Middle-R	Aacatgcttagtgatttggcgagt	
dCas9-DOWN-F	actcgccaaatcactaagcatgtTgcacaaattttggatagtcgcat	
dCas9-DOWN-R	tccgcctgtctgtacttctgttttc	
P_{trc}-F	gaaaacagaagtacagacaggcggagcgcaacgcaattaatgtgag	
P_{trc}-R	ccacacattatacgagccggat	
cas9_S_4-F	ctcgtataatgtgtggtcggcacaaatagcgtcggagttttagagctagaaatagcaag	
BIRI-R	ctcggtacccggggatccgtagggataacagggtaatagat	
P_{H36}-F	gaaaacagaagtacagacaggcggattcaccgtcatcaccgaaacg	pP_{H36}sgRNA-dCas9
P_{H36}-R	GGATCCCATGCTACTCCTACCAA	
cas9_S_110-F	GAGTAGCATGGGATCCtcgccaaatcactaagcatggttttagagctagaaatagcaag	

BIRI-R	ctcggtacccggggatccgtagggataacagggtaataga t	
19ts-XXH-F	GATCCCCGGGTACCGAGCT	$pP_{trc}P_{H36}$sgRNA-d Cas9
19ts-XXH-R	CTGTTGCCCGTCTCACTGGTG	
P_{trc}sgRNA-F	caccagtgagacgggcaacaggcgcaacgcaattaatgt gag	
P_{trc}sgRNA-R	agagtttgtagaaacgcaaaaaggc	
dCas9-UP-F	gcctttttgcgtttctacaaactcttaaacacccagagcctgt cgc	
BIRI-R	ctcggtacccggggatccgtagggataacagggtaataga t	
aspA-UP-F2	cgacggccagtgccaagcttccatcgccgatgttgtatcca c	pK18-P_{tuf}-aspB_P a
aspA-UP-R2	tctaacaagccgctaccccacttaccctacgtactgcctctc acaagttgaaggaa	
P_{tuf}-F	GTAGGGTAAGTGGGGTAGCGG	
P_{tuf}-R	TGTATGTCCTCCTGGACTTCGTG	
aspB_Pa-F	cacgaagtccaggaggacatacaatgctgaacattgtgat gatcgg	
aspB_Pa-R	aattgcccaggtatcagacgagatcttggattagattgaaat ggcatgggca	
aspA-DOWN-F	tccaagatctcgtctgatacctgg	
aspA-DOWN-R	agctcggtacccggggatccgcctctgaaacggcgaagc t	

gltA-UP-F	aacgacggccagtgccaagcttatggctaaaccgcatttat cgg	pK18-gltA-QC
gltA-UP-R	cctcgttgaaggtggcattctc	
gltA-DOWN-F	gagaatgccaccttcaacgaggccaggctgttctggagat gctc	
gltA-DOWN-R	agctcggtacccggggatccctggcgtgggcggttgat	
gltA(P)-UP-F	cgacggccagtgccaagcttgcggggattagttgaccagc	pK18-$P_{NCgl2698}$-glt A
gltA(P)-UP-R	cttagattctgtgcttttaagcagagactcttccggattacgg aagtagtcc	
cspA ter-F	TCTCTGCTTAAAAGCACAGAATCTA AG	
csp ter-R	GAGTCTTCGGCGGGAAATTATTC	
$P_{NCgl2698}$-F	gaataatttcccgccgaagactcTTTCTAGCAAA TTAAGCGGGCA	
$P_{NCgl2698}$-R	TGGGTCTCCTTTGGGCCAC	
gltA(P)-DOWN-F	GTGGCCCAAAGGAGACCCAatgtttgaaa gggatatcgtggct	
gltA(P)-DOWN-R	agctcggtacccggggatcccgagtgggttcagctggtcc t	
gltA(P)-UP-F	cgacggccagtgccaagcttgcggggattagttgaccagc	pK18-P_{iolT1}-gltA
gltA(P)-UP-R	cttagattctgtgcttttaagcagagactcttccggattacgg aagtagtcc	
cspA ter-F	TCTCTGCTTAAAAGCACAGAATCTA AG	

csp ter-R	GAGTCTTCGGCGGGAAATTATTC	
P_{iolT1}-F	gaataatttcccgccgaagactctcaccccctgaacccgcct	
P_{iolT1}-R	cttgtctcctaagtttgtcgtgcc	
gltA(P)-DOWN-F	acgacaaacttaggagacaagatgtttgaaagggatatcgtggct	
gltA(P)-DOWN-R	agctcggtacccggggatcccgagtgggttcagctggtcct	
recEC-cas9-DOWN-F	aacaggaagagcccgtaaacctc	$pP_{trc}P_{H36}$sgRNA-recET-cas9
recEC-cas9-DOWN-R	tggtcaaagcttcccctggag	
XXH-F	ctccaggggaagctttgaccattcaccgtcatcaccgaaacg	
XXH-R	gaggtttacgggctcttcctgtttaagcctattgagtatttcttatccataatc	

Table S2. The primers used for construction of sgRNA plasmid.

Primers	Sequences (5'-3')*
P_{glyA}2-F	agacagaacttaatgggcccAGCTACTCCACTAGTGTGATCGGGG
P_{glyA}2-R	gcccacaagcatagacCGCC
Null-sgRNA-F	gtctatgcttgtgggc*TTCAAGAGCGCCATGCCTGA*GTTTTAGAGCTAGAAATAGCAAG
BIRI2-R	ctcggtacccggggatcCGTAGGGATAACAGGGTAATAGAT
mCherry-sgRNA-F	gtctatgcttgtgggc*CAAGGGCGAGGAGGATAACA*GTTTTAGAGCTAGAAATAGCAAG
ptsG-sgRNA-F	gtctatgcttgtgggc*GACGACATCGCAACATATTC*GTTTTAGAGCTAGAAATAGCAAG
ptsH-sgRNA-F	gtctatgcttgtgggc*GCTTCCAAGACTGTAACCGT*GTTTTAGAGCTAGAAATAGCAAG
ptsI-sgRNA-F	gtctatgcttgtgggc*GAATCAAGACACTGTACTGA*GTTTTAGAGCTAGAAATAGCAAG
iolR-sgRNA-F	gtctatgcttgtgggc*TGACCACCGAAGCTCCCATT*GTTTTAGAGCTAGAAATAGCAAG
pgi-sgRNA-F	gtctatgcttgtgggc*ACATTTCGACCACCCAGGTT*GTTTTAGAGCTAGAAATAGCAAG
pfk-sgRNA-F	gtctatgcttgtgggc*TTTGGGTGATTGTTCCGGCG*GTTTTAGAGCTAGAAATAGCAAG
gapA-sgRNA-F	gtctatgcttgtgggc*CGTGTTGGTATTAACGGATT*GTTTTAGAGCTAGAAATAGCAAG
gapB-sgRNA-F	gtctatgcttgtgggc*CGACCTGCGTGCCTTCCTGG*GTTTTAGAG

	CTAGAAATAGCAAG
zwf-sgRNA-F	gtctatgcttgtgggc*CCTTCCGGCATGGTGATCTT*GTTTTAGAGCTAGAAATAGCAAG
tktA-sgRNA-F	gtctatgcttgtgggc*GACGCTGTCACCTGAACTTC*GTTTTAGAGCTAGAAATAGCAAG
pgl-sgRNA-F	gtctatgcttgtgggc*CGCACGCGATACTGAAGATT*GTTTTAGAGCTAGAAATAGCAAG
gnd-sgRNA-F	gtctatgcttgtgggc*GGAGATAATCTCGCACAGAT*GTTTTAGAGCTAGAAATAGCAAG
NCgl2337-sgRNA-F	gtctatgcttgtgggc*ACTTCCATGCGCGTATACCT*GTTTTAGAGCTAGAAATAGCAAG
gltA-sgRNA-F	gtctatgcttgtgggc*CGTGGCTACTGATAACAACA*GTTTTAGAGCTAGAAATAGCAAG
mdh-sgRNA-F	gtctatgcttgtgggc*GCAGAACGTCTCCACCAAGA*GTTTTAGAGCTAGAAATAGCAAG
mqo-sgRNA-F	gtctatgcttgtgggc*GAGGCAGATGTAGTTCTCAT*GTTTTAGAGCTAGAAATAGCAAG
kgd-sgRNA-F	gtctatgcttgtgggc*GTGAGCAGCGCTAGTACTTT*GTTTTAGAGCTAGAAATAGCAAG
aceF-sgRNA-F	gtctatgcttgtgggc*CTGGGCGAATCAGTAACCGA*GTTTTAGAGCTAGAAATAGCAAG
lpd-sgRNA-F	gtctatgcttgtgggc*GACGTAGTAGTACTCGGAGC*GTTTTAGAGCTAGAAATAGCAAG
sucC-sgRNA-F	gtctatgcttgtgggc*CGGGACCTCTTTGAAACCCA*GTTTTAGAGCTAGAAATAGCAAG

NCgl2476-sgRNA-F	gtctatgcttgtgggc*AGATTCCCGCATCATCATTC*GTTTTAGAGCTAGAAATAGCAAG
NCgl2480-sgRNA-F	gtctatgcttgtgggc*TGAAAAAGTCCGATTACCTG*GTTTTAGAGCTAGAAATAGCAAG
aceE-sgRNA-F	gtctatgcttgtgggc*ATGGCCGATCAAGCAAAACT*GTTTTAGAGCTAGAAATAGCAAG
ldh-sgRNA-F	gtctatgcttgtgggc*GGTAACAAGATTGTCCTCAT*GTTTTAGAGCTAGAAATAGCAAG
poxB-sgRNA-F	gtctatgcttgtgggc*ATAACGTTGAGGAGTTCAGA*GTTTTAGAGCTAGAAATAGCAAG
ackA-sgRNA-F	gtctatgcttgtgggc*ATCTTCCATCAAATTCCAGC*GTTTTAGAGCTAGAAATAGCAAG
pta-sgRNA-F	gtctatgcttgtgggc*GACCTCAGCTCTGATCACCA*GTTTTAGAGCTAGAAATAGCAAG
NCgl1987-sgRNA-F	gtctatgcttgtgggc*TTTGTTCATGGTCATGTCCA*GTTTTAGAGCTAGAAATAGCAAG
alaA-sgRNA-F	gtctatgcttgtgggc*ACAAAGTGGCAACATTGAGC*GTTTTAGAGCTAGAAATAGCAAG
NCgl2247-sgRNA-F	gtctatgcttgtgggc*CATTCTTCTGGTTTAGGCAC*GTTTTAGAGCTAGAAATAGCAAG
NCgl0670-sgRNA-F	gtctatgcttgtgggc*CGAGACTAGGAAGATCACCA*GTTTTAGAGCTAGAAATAGCAAG
NCgl2309-sgRNA-F	gtctatgcttgtgggc*CGGCGTCCCTGTTGAAGAAT*GTTTTAGAGCTAGAAATAGCAAG
NCgl0245-sgRNA-F	gtctatgcttgtgggc*TAACCGCTACATGCCTTTCG*GTTTTAGAGCT

	AGAAATAGCAAG
purA-sgRNA-F	gtctatgcttgtgggc*ATGGCTGCAATCGTTATTGT*GTTTTAGAGCT AGAAATAGCAAG
argG-sgRNA-F	gtctatgcttgtgggc*CGCATCGTTCTTGCATACTC*GTTTTAGAGCT AGAAATAGCAAG

*：The lowercase parts were overlap; The italics were N20 sequences.

Table S3. The primers used for quantitative PCR.

Primer	Primer sequence (5'-3')
16 s rDNA-F	GGTGATCTGCCCTACACTTT
16 s rDNA-R	CCATTACCCCACCAACAA
gapA-qPCR-F	TGGTATTAACGGATTTGGC
gapA-qPCR-R	TGGAGTCATCGTCGTATTCA
gapB-qPCR-F	GCTGCGTCGTGATTCTGT
gapB-qPCR-R	GATGCCGTATTCGGTGTAA
gltA-qPCR-F	TCGTGGCTACTGATAACAACA
gltA-qPCR-R	ACATAACCTGGGTCAAAAGTG
